# Supplementary material for: Scaffolding Early Dialogue: A Unified Account of Response Contingency in Child–Caregiver Interaction
Source: Cogn Sci. 2026 Jul 26;50(7):e70245. doi: 10.1111/cogs.70245 (PMC13401803; doi:10.1111/cogs.70245)
Supplement: Supplementary file 1 — Supporting Information [file COGS-50-e70245-s001.pdf]

Appendix A

Summary statistics of the dataset

This appendix provides corpus-level summary statistics for the 40 corpora included in the analysis. Tables A1 and A2 report, for each corpus, the number of transcripts, the total number of child responses, the median child age, the mean length of utterance (MLU), the average contingency score, the number of distinct communicative intents, and intent entropy (ranging from 0, indicating that a single intent dominates, to 1, indicating a maximally diverse distribution of intents). These descriptive statistics are included to document the composition and variability of the corpora used in the study.

Table A1

Summary statistics for the corpora (Part 1)

Corpus	Transcripts	Responses	Median age	MLU child	MLU caregiver	Cont. child	Cont. caregiver	Intents child	Intents caregiver	Entropy child	Entropy caregiver
Bates	100	3047	28	1.88	3.72	0.68	0.78	22	20	0.82	0.80
Belfast	15	903	31	3.16	5.82	0.49	0.56	14	13	0.81	0.76
Bloom	24	15099	28	2.87	5.19	0.57	0.75	32	20	0.73	0.79
Braunwald	438	6935	26	2.69	3.87	0.46	0.67	27	32	0.76	0.76
Brown	51	13363	28	2.51	4.17	0.51	0.81	30	38	0.75	0.71
Clark	39	2066	37	3.23	4.69	0.58	0.74	20	25	0.76	0.74
Cornell	43	2300	29	2.50	4.00	0.56	0.73	17	22	0.78	0.77
Cruttenden	48	1056	27	1.90	3.91	0.52	0.72	13	16	0.74	0.73
Davis	259	4871	24	2.39	3.81	0.49	0.68	22	28	0.78	0.78
Demetras1	65	1154	30	2.86	4.71	0.53	0.71	14	15	0.80	0.75
Demetras2	130	3256	37	3.51	5.11	0.63	0.73	18	16	0.78	0.73
Evans	94	2602	25	2.14	3.62	0.51	0.66	20	22	0.79	0.77
Feldman	25	3530	32	3.32	5.18	0.59	0.73	23	18	0.76	0.76
Garvey	36	2756	35	3.64	5.01	0.57	0.75	20	18	0.76	0.74
Gathercole	58	2095	26	2.19	3.80	0.48	0.68	18	21	0.80	0.78
Gleason	42	1367	27	2.20	3.84	0.47	0.69	16	19	0.79	0.77
Hall	37	2322	30	2.79	4.61	0.54	0.72	21	18	0.77	0.76
Higginson	64	1792	29	2.46	4.15	0.50	0.70	18	20	0.79	0.76
Howe	52	1475	26	2.08	3.70	0.49	0.68	17	19	0.80	0.78
Korman	110	3940	33	3.08	4.85	0.56	0.74	22	20	0.77	0.75

Note. MLU = mean length of utterance. Median age is in months. Cont. = average contingency score. Intents = number of distinct communicative intents. Entropy refers to the entropy of communicative intents.

Table A2

Summary statistics for the corpora (Part 2)

Corpus	Transcripts	Responses	Median age	MLU child	MLU caregiver	Cont. child	Cont. caregiver	Intents child	Intents caregiver	Entropy child	Entropy caregiver
Lara	74	2210	24	2.04	3.66	0.50	0.67	19	22	0.79	0.77
MacWhinney	55	2604	31	2.91	4.57	0.55	0.72	21	19	0.78	0.75
McCune	40	1188	23	1.82	3.45	0.47	0.66	14	18	0.81	0.79
Morisset	97	2861	30	2.74	4.42	0.54	0.71	20	18	0.77	0.75
Nelson	44	1520	28	2.35	3.98	0.51	0.69	17	21	0.79	0.77
NewEngland	122	4517	29	2.67	4.31	0.53	0.72	21	20	0.78	0.76
Peters	68	1734	25	2.11	3.73	0.49	0.68	18	21	0.80	0.78
Post	83	2458	27	2.41	4.06	0.52	0.70	19	20	0.79	0.76
Sachs	58	1974	26	2.23	3.88	0.50	0.69	18	20	0.80	0.77
Sawyer	46	1211	24	1.96	3.61	0.48	0.67	15	18	0.81	0.79
Snow	71	2543	30	2.78	4.39	0.55	0.73	20	19	0.77	0.75
Soderstrom	63	1830	28	2.32	3.95	0.51	0.70	18	20	0.79	0.77
Suppes	95	3289	31	2.89	4.60	0.56	0.73	21	19	0.77	0.75
Tardif	87	2417	29	2.58	4.22	0.53	0.71	20	20	0.78	0.76
Valian	49	1426	25	2.09	3.68	0.49	0.68	17	19	0.80	0.78
VanHouten	53	1598	27	2.27	3.90	0.50	0.69	18	20	0.79	0.77
Warren	60	1887	28	2.38	4.03	0.52	0.70	19	20	0.79	0.76
Weist	41	1175	24	1.94	3.58	0.48	0.67	15	18	0.81	0.79
Wells	77	2364	30	2.72	4.36	0.54	0.72	20	19	0.78	0.75
Wilson	69	2099	29	2.49	4.11	0.52	0.71	19	20	0.79	0.76

Note. MLU = mean length of utterance. Median age is in months. Cont. = average contingency score. Intents = number of distinct communicative intents. Entropy refers to the entropy of communicative intents.

Appendix B

Lexical frames used by caregivers

This appendix provides illustrative examples of the most frequent lexical frames identified in the data. For each frame length, Tables B1 (N=1, unigrams), B2 (N=2, bigrams), and B3 (N=3, trigrams) report the five most dominant communicative intents, the number of distinct frames associated with each intent, their total frequency, and representative frame examples with corresponding occurrence counts. These materials are included to give a concrete sense of the lexical patterns underlying the caregiver form-function regularity. Throughout these tables, we use the same intent labels as Snow et al. (1996).

Table B1

Top frames for Frame Length = 1 (Unigram)

Intent	# Frames	Total Occ.	Top frames (occurrence)
QN (Wh- Question)	7	36166	what (22625) | where (5992) | who (3021) | why (1869) | how (1787) which (672) | whose (200)
YQ (Yes/no Question)	13	18784	is (6764) | are (4161) | did (2718) | does (1138) | have (1088) pardon (1078) | has (519) | was (469) | really (207) | were (193) remember (183) | again (138) | hum (128)
RP (Request/Suggest an action)	12	8174	let (1999) | come (1949) | look (1843) | put (1023) | sit (290) turn (207) | give (197) | try (162) | show (155) | listen (120) push (119) | pull (110)
MK (Social norms)	8	4190	thank (1411) | hello (815) | hi (453) | bye (446) | oops (427) whoops (225) | byebye (213) | whoa (200)
AC (Show attentiveness)	2	3793	mhm (3314) | uhhum (479)

Note. Intent = top communicative intent; # Frames = number of distinct frames; Total Occ. = total occurrences across those frames.

Table B2

Top frames for Frame Length = 2 (Bigram)

Intent	# Frames	Total Occ.	Top frames (occurrence)
QN (Wh- Question)	43	27868	what is (8174) | where is (3218) | what are (2103) | what do (1724) | who is (1635)<br>and what (1196) | what did (944) | what color (610) | what does (597) | oh what (492)<br>where are (473) | what else (437) | what happened (376) | where did (368) | how many (367)<br>what you (343) | which one (336) | what kind (290) | and who (279) | where does (262)<br>where do (259) | what re (234) | what have (222) | what was (218) | why not (192)<br>what would (184) | why are (183) | where has (173) | why is (155) | how come (150)
YQ (Yes/no Question)	35	23907	do you (4249) | are you (3245) | is that (2301) | is it (1862) | you want (1781)<br>you do (1756) | did you (1664) | is he (741) | have you (739) | you going (549)<br>are they (421) | you like (386) | oh is (343) | is there (309) | is she (300)<br>you think (287) | does it (282) | is the (238) | does he (202) | did he (184)<br>is not (174) | was it (173) | does that (164) | is this (164) | oh are (152)<br>has he (147) | are we (130) | were you (130) | and did (129) | has it (128)
ST (Statement)	36	10791	there is (1983) | oh that (793) | this is (759) | here is (671) | i can (482)<br>we have (445) | i have (426) | i see (403) | and the (353) | and that (325)<br>and we (282) | i thought (264) | he has (220) | oh he (219) | i did (203)<br>and i (198) | well it (185) | and there (180) | uh huh (168) | it looks (163)<br>and this (158) | it has (153) | and he (151) | she has (144) | oh she (143)<br>these are (141) | they have (140) | that looks (125) | there are (125) | and it (122)
RP (Request/Suggest an action)	12	5544	let us (1493) | come on (1145) | look at (613) | oh look (446) | come here (417)<br>let me (351) | put it (308) | go on (255) | put the (149) | sit down (139)<br>go and (127) | turn it (101)
MK (Social norms)	6	3675	thank you (1407) | oh dear (1020) | there we (514) | oh thank (275) | bye bye (235)<br>uh oh (224)

Note. Intent = top communicative intent; # Frames = number of distinct frames; Total Occ. = total occurrences across those frames.

Table B3

Top frames for Frame Length = 3 (Trigram)

<table>
<tr><th>Intent</th><th># Frames</th><th>Total Occ.</th><th>Top frames (occurrence)</th></tr>
<tr><td>QN (Wh- Question)</td><td>43</td><td>17008</td><td>what is that (2539) | what are you (1516) | what do you (1453) | where is the (1145) | what is this (999)
what is it (961) | what is the (886) | what did you (607) | who is that (515) | and what is (412)
what is he (403) | where is your (346) | what color is (259) | what kind of (252) | where is it (249)
where are you (244) | oh what is (238) | what is in (225) | what you doing (196) | where do you (195)
what are they (195) | what re you (195) | what is wrong (190) | what is she (189) | what have you (174)
who is it (158) | what is what (157) | where did you (154) | why are you (154) | and who is (152)</td></tr>
<tr><td>YQ (Yes/no Question)</td><td>31</td><td>9335</td><td>you do not (1284) | do you want (946) | are you going (817) | you are going (699) | you going to (532)
is that a (474) | do you think (430) | do you like (342) | you want me (339) | is that what (241)
do you remember (230) | is it a (229) | is that the (209) | you want a (191) | you wanna go (186)
is that your (170) | you want the (168) | you want some (161) | did you see (158) | are you a (152)
have you got (151) | is there a (148) | do you have (135) | oh is that (128) | oh you want (127)
oh you wanna (125) | oh are you (118) | are you sure (116) | you wanna get (115) | did you have (110)</td></tr>
<tr><td>ST (Statement)</td><td>27</td><td>5205</td><td>oh that is (633) | there is a (625) | i can not (276) | i think you (263) | i am not (253)
there is the (246) | and that is (208) | it is the (203) | this is a (188) | here is a (159)
and then we (148) | oh there is (146) | it does not (145) | i did not (142) | and there is (140)
i thought you (132) | here is the (132) | it looks like (128) | we do not (128) | i have got (123)
oh you have (120) | well it is (119) | there is no (118) | he is a (114) | you have not (111)
well i think (105) | oh i think (100)</td></tr>
<tr><td>AP (Agree with proposition)</td><td>5</td><td>3297</td><td>that is right (2268) | yeah that is (431) | yeah it is (236) | yes that is (190) | yes it is (172)</td></tr>
<tr><td>RP (Request/Suggest an action)</td><td>12</td><td>2159</td><td>why do not (407) | can you see (305) | come on then (272) | go on then (168) | let me see (153)
can you put (141) | look at this (126) | let us go (126) | oh look at (125) | let us have (120)
look at that (112) | look at the (104)</td></tr>
</table>

Note. Intent = top communicative intent; # Frames = number of distinct frames; Total Occ. = total occurrences across those frames.

Appendix C

Interactive Routines

This appendix documents the interactive routines used in the analysis (Table C1). Table C2 provides illustrative excerpts from the New England corpus for each routine type. Throughout these tables, we use the same intent labels as Snow et al. (1996).

Table C1

The list of interactive routines.

Initiating intent	Responding intent
YQ (Yes/No Question)	AA (Affirmative answer to Yes/No Question)
YQ (Yes/No Question)	AN (Negative answer to Yes/No Question)
QN (Wh- Question)	SA (Wh- Answer)
RQ (Yes/No Suggestion)	AD (Agree to suggested act)
RQ (Yes/No Suggestion)	RD (Refuse suggested act)
RP (Request/Suggest an action)	AD (Agree to suggested act)
RP (Request/Suggest an action)	RD (Refuse suggested act)

Table C2

Excerpts from the New England corpus illustrating each routine sequence defined in Table C1.

Initiation	Excerpt (caregiver)	Response	Excerpt (child)
YQ	*do you want both monsters?*	AA	*yep.*
YQ	*you don't?*	AN	*no.*
QN	*what's this?*	SA	*beach.*
RQ	*should we get another box?*	AD	*yeah.*
RQ	*oh do you want to draw pictures?*	RD	*I don't.*
RP	*turn around this way*	AD	*okay.*
RP	*could you get me that box?*	RD	*no it's too heavy.*

Appendix D

Annotation guidelines for response contingency

This Appendix summarizes the coding scheme used by Agrawal et al. (2024) for the manual annotation of response contingency in the New England corpus. Annotators labeled contingency in both children's and caregivers' turns. Annotations were based solely on the linguistic context available in the transcript (i.e., without access to multi-modal information or any additional labels).

Only utterances occurring at turn switches were annotated, that is, pairs of turns in which one speaker initiates and the other responds. Consecutive utterances produced by the same speaker were therefore not coded for (intra-speaker) contingency. Nevertheless, annotators were allowed to consider the broad conversational context—including preceding and subsequent turns—when needed to disambiguate a given exchange. Each turn switch was marked as contingent (+1) or non-contingent (-1). In cases of uncertainty—when the transcript did not provide sufficient information to determine contingency—the label 0 (ambiguous) was assigned. Below we provide examples of particularly challenging cases that required annotators to establish shared guidelines.

One of these cases is **topic transitions**. These were evaluated on a case-by-case basis:

- *Smooth transitions* that reflected a natural progression of the ongoing activity were coded as contingent. Table D1 shows an example in which the caregiver slightly shifts the topic by suggesting a new action, while remaining contingent on the prior exchange.

- *Abrupt transitions* that did not clearly follow from the ongoing activity were coded as non-contingent. Table D2 presents an example in which the caregiver abruptly redirects the child's attention to a new topic; this turn was therefore marked as non-contingent.

- When the distinction between smooth and abrupt could not be determined from

the transcript alone, the turn was labeled 0.

Another case is that of **short vocalizations** such as “oh”, “mhm”, “mm”, and “uh-huh”. They were likewise evaluated on a case-by-case basis:

- Table D3 illustrates an example in which a short vocalization (here, “Oh”) was coded as contingent because a subsequent utterance by the same speaker retrospectively clarified its contingent function.
- Table D4 presents an excerpt in which the contingency of “oh” could not be determined from the available context. The same excerpt shows that another short vocalizaton “mhm” was coded as contingent, as it functioned here as a response to a yes–no question.

Other annotation guidelines: when a question is produced in response to another question it was marked as -1 unless it functioned as a clarification request. Clarification requests were marked as +1. Finally, redundant utterances and unclear vocalizations were labeled 0.

Table D1

Example for smooth topic transition.

Speaker code	Transcript	Annotation
MOT	what is it?	
MOT	a book!	
CHI	yeah.	1
MOT	oh you want me to read it?	1

Table D2

Example for abrupt topic transition.

Speaker code	Transcript	Annotation
MOT	what are you going to do now?	
CHI	going to do.	1
MOT	what's that?	-1
MOT	is that a block?	
MOT	is that a block?	

Table D3

Example for short vocalization.

Speaker code	Transcript	Annotation
MOT	what's this?	
MOT	what's in this box?	
CHI	oh.	1
CHI	oh this.	

Table D4

Examples for short vocalization.

Speaker code	Transcript	Annotation
MOT	yeah.	
MOT	What's that?	
CHI	oh.	0
MOT	is that a cow?	
CHI	mhm.	1

Appendix E

Model details for automatic annotation of communicative intents

This appendix describes the model that we used to automatically annotate the English-language CHILDES corpora for communicative intents as developed by Nikolaus et al. (2022). The model is a Conditional Random Field (CRF) model and its implementation is based on *pycrfsuite*[4].

A CRF is a probabilistic sequence-labeling model that exploits contextual information to predict labels. Unlike classifiers that treat each utterance independently, a CRF can model dependencies among neighboring labels in a sequence. In the present case, this allows the model to learn dependencies among the 67 communicative intent categories in the INCA-A coding scheme and to capture common conversational patterns. For example, a yes/no question is often followed by an affirmative or negative answer, and the CRF can use such regularities to improve intent prediction.

For each utterance, the CRF model has access to a set of features, including speaker identity (child or caregiver), lexical features such as unigrams and bigrams, part-of-speech tags, and word repetitions with the preceding utterance. To illustrate how the model operates, consider the following exchange between a caregiver and a child:

Caregiver: Do you want both monsters?

Child: Yep.

To identify the communicative intents expressed in these two utterances, the CRF model must assign an intent label to each turn. Figure E1 illustrates the dependencies exploited by the model in this example. Formally, given a sequence of observed states $X = \{x_1, x_2, x_3, \ldots, x_n\}$ and corresponding labels $Y = \{y_1, y_2, y_3, \ldots, y_n\}$, the CRF learns a mapping from observations to labels, where each label corresponds to one of the 67 communicative intents defined in the coding scheme.

For the first utterance by the caregiver which corresponds to the observed state

[4] https://github.com/scrapinghub/python-crfsuite

x_1, the model receives the following set of features: a list of unigrams, bigrams , POS tags, speaker identity and word repetitions as shown in the figure. Now rather than classifying each utterance in isolation, it looks at the entire conversation flow as a chain and assigns the most likely sequence of intent labels jointly. Given that the bigram "do you" is highly diagnostic of the YQ (Yes/No Question) intent as it occurs frequently in the corpus with this context, the CRF model correctly tags the utterance with the label YQ as it begins with this bigram.

For the second utterance, the model has learned during training that a Yes/No Question (YQ) is typically followed by either an Affirmative Answer (AA) or a Negative Answer (AN). It therefore uses this expectation to narrow the set of plausible labels for the response turn. Combined with the fact that the unigram "yep" is strongly associated with affirmative answers in the training data, the model correctly assigns the label AA to the child's utterance.

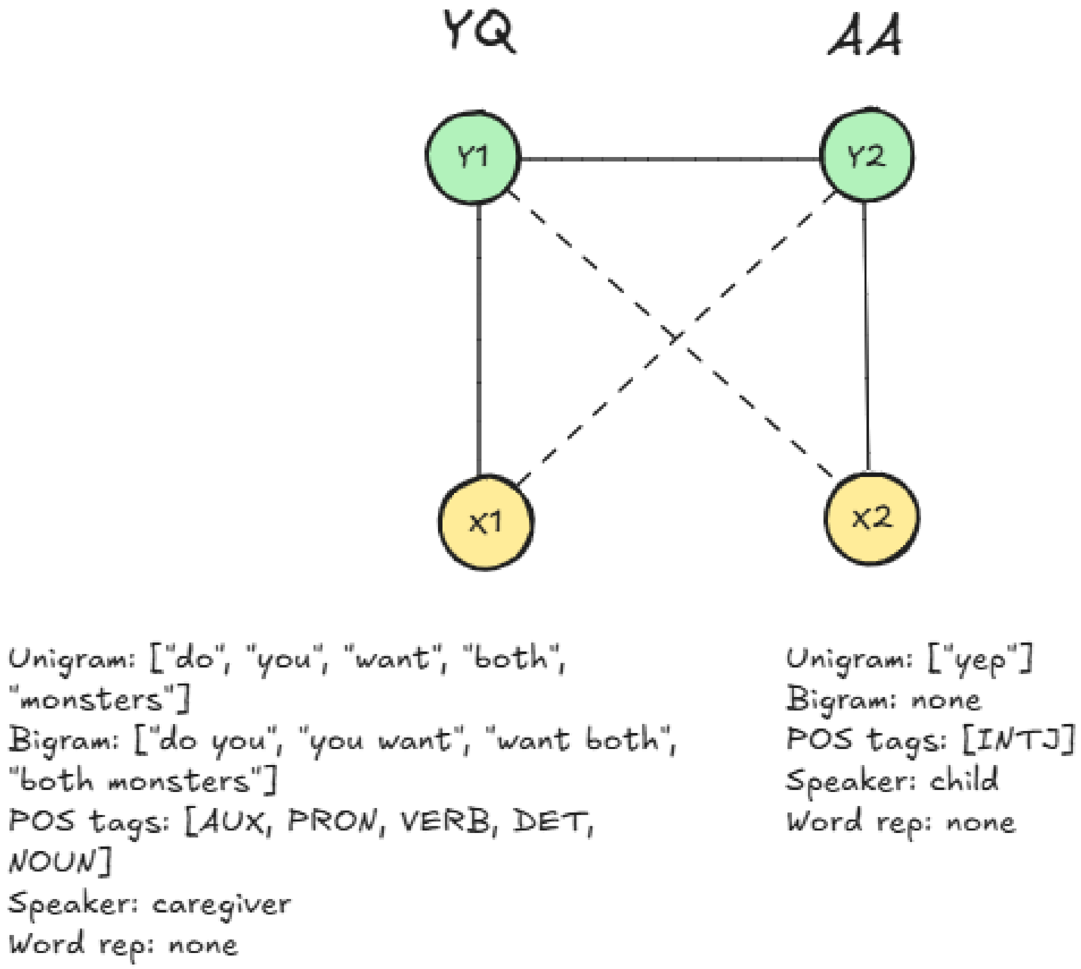

Figure E1

A representation of the connections between the observed states and the labels for a CRF model.

Appendix F

Model details for automatic annotation of response contingency

This appendix describes the model used to automatically annotate response contingency in the English-language CHILDES corpora, following the approach of Agrawal et al. (2024). We used DeBERTaV3 (He et al., 2022) as the backbone model and fine-tuned it in a supervised manner to predict the coherence of a turn given its conversational context, defined as the five preceding turns.

There are two phases in training the model: pre-training and fine-tuning. We did not perform the pre-training ourselves; instead, we used the version released by the model's authors. In the fine-tuning phase, we adapted this pre-trained model to perform contingency classification. Figure F1 shows a representation of the pre-training phase of the model with an example.

Pre-training phase. DeBERTaV3 is pre-trained using a Replaced Token Detection (RTD) objective. In this setup, a generator first replaces some masked tokens in the input sequence with plausible alternatives. A discriminator is then trained to determine, for each token, whether it is an original token or one that has been replaced by the generator. In the example shown in Figure F1, the token "bird" is replaced by "dog". The discriminator must therefore learn that "dog" is the only replaced token, while the remaining tokens are original. DeBERTaV3 also uses disentangled attention, which represents each token in terms of both content and relative position information and computes attention over these components separately. Together, the RTD objective and disentangled attention allow the model to learn rich contextual representations. In the present work, we do not pre-train DeBERTaV3 ourselves; instead, we use the publicly released pre-trained model as the starting point for fine-tuning.

Fine-tuning phase. Once the model has been pre-trained, it can be fine-tuned for a downstream task. During fine-tuning, only the discriminator is retained and further trained, while the generator is discarded. Figure F2 illustrates this phase. The model receives the current turn together with the five preceding turns as conversational context and is trained to classify the current turn as contingent, non-contingent, or

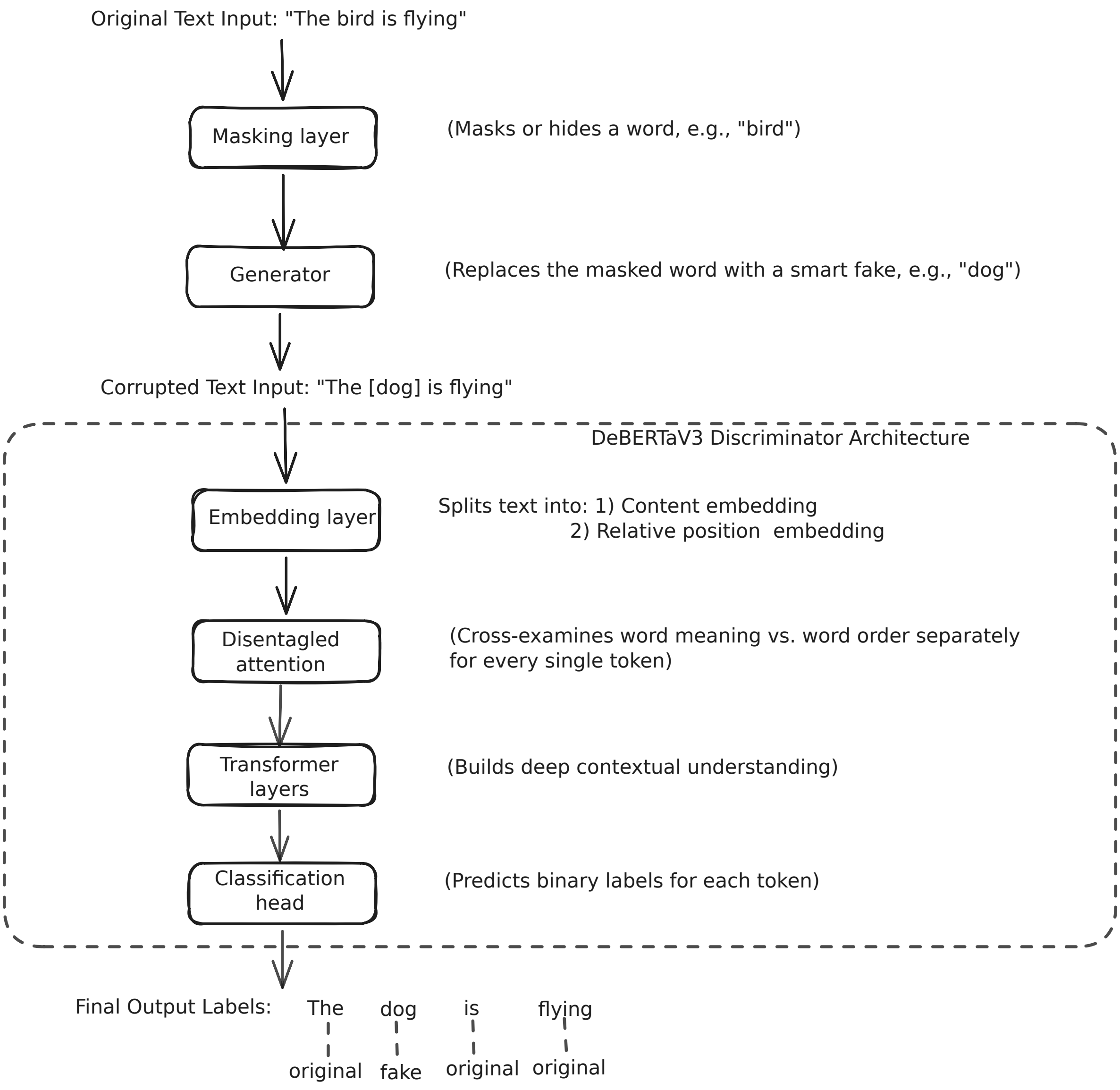

Figure F1

A representation of the pre-training phase of the DeBERTaV3 model.

ambiguous with respect to that context. This process leverages the contextual representations acquired during pre-training, which are then adapted to the requirements of the specific task.

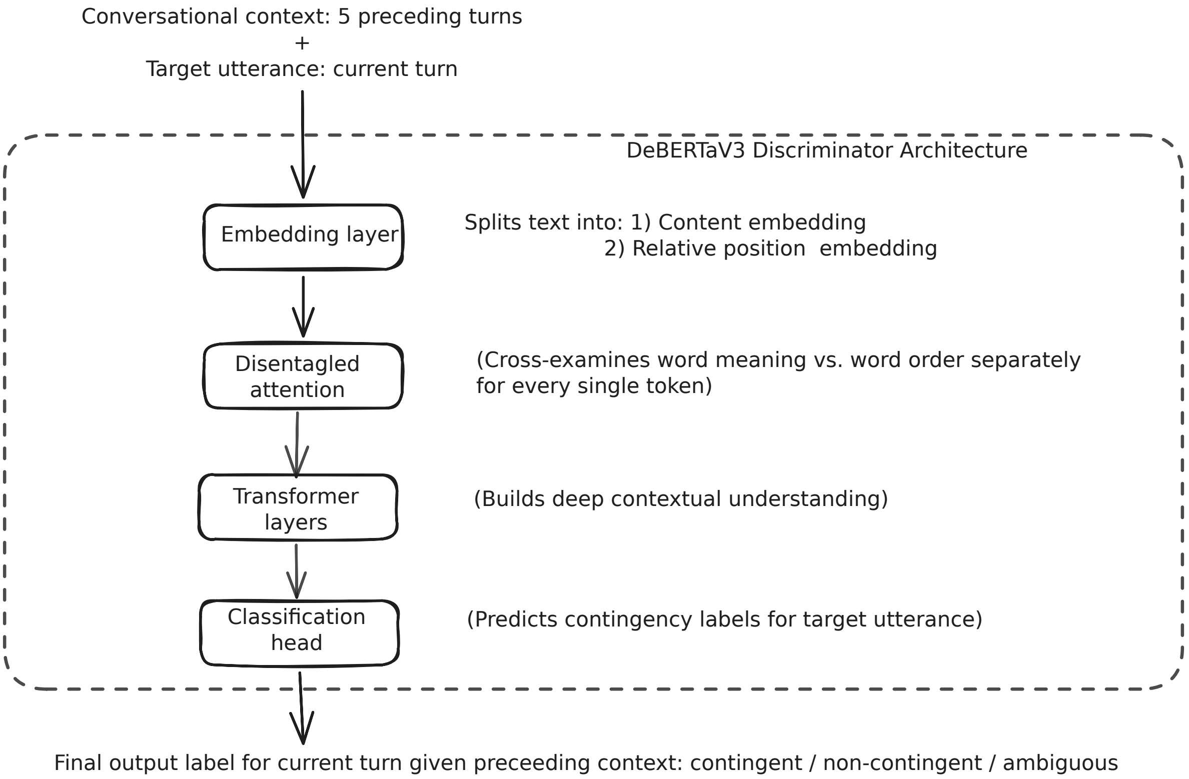

Figure F2

A representation of the fine-training phase of the DeBERTaV3 model.
